# The 130 to 375 GHz Rotational Spectroscopy of *s*-*trans*-(*Z*)‑1-Cyano-1,3-butadiene (C_5_H_5_N): Analysis of the Lowest-Energy Vibrationally Excited Dyad (ν_19_ and ν_27_)

**DOI:** 10.1021/acs.jpca.5c04066

**Published:** 2025-09-04

**Authors:** P. Matisha Dorman, Brian J. Esselman, Andrew N. Owen, R. Claude Woods, Robert J. McMahon

**Affiliations:** Department of Chemistry, 5228 University of Wisconsin−Madison, Madison, Wisconsin 53706, United States

## Abstract

The rotational spectra and analysis of the two lowest-energy vibrationally excited states, ν_19_ (A′, 126 cm^–1^, MP2) and ν_27_ (A″, 133 cm^–1^, MP2), of *s-trans-(Z)*-1-cyano-1,3-butadiene from 130 to 375 GHz is presented. The state symmetries allow *a-* and *b-*type Coriolis coupling, the effects of which are observed due to the close energy spacing of these states. A combined total of 6744 transitions were modeled (σ_fit_ < 60 kHz) with a partial-octic two-state A-reduced Hamiltonian including eight coupling parameters (*G*_*a*_, *G*_*a*_^*J*^, *F*_*bc*_, *F*_*bc*_^*J*^, *G*_*b*_, *G*_*b*_^*J*^, *G*_*b*_^*JJ*^, and *G*_*b*_^*K*^). The vibration–rotation interaction constants, Coriolis ζ terms, and energy difference between the states were determined experimentally and are compared to predicted values (B3LYP/6-311+G­(2d,p) and MP2/6-311+G­(2d,p)). The coupled-state analysis affords a precise vibrational energy difference of 5.753 373 1 (25) cm^–1^ between the two fundamental states. Several matching pairs of *a*- and *b*-type resonant transitions, as well as many formally forbidden, nominal interstate transitions, are included in the final least-squares fit. (*E*)- and (*Z*)-1-Cyano-1,3-butadiene are possible precursors to or degradation products of pyridine, a fundamental aromatic molecule of interest in the interstellar medium. There is currently some uncertainty concerning the reported identifications of other cyanobutadiene isomers in the interstellar medium. Combined with our previously reported ground-state spectroscopic constants, the vibrationally excited-state constants provided herein enable possible extraterrestrial identification of multiple vibrational states of *s-trans-(Z)*-1-cyano-1,3-butadiene.

## Graphical abstract

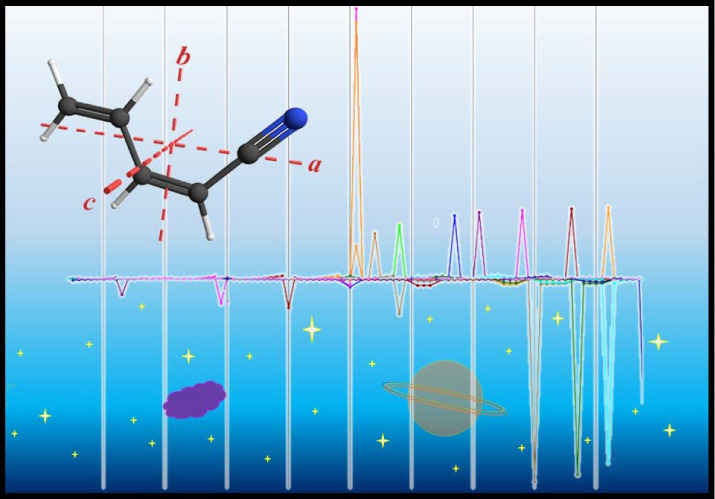

## Introduction

An interesting variety of unsaturated, nitrile-containing organic molecules, including benzonitrile,^1^ cyanonaphthalenes,^2^ cyano-1,3-cyclopentadienes,^3,4^ allenylcyanoacetylene (H_2_CCCH­(CC)­CN),^5^ methyl­(cyanotriacetylene) (CH_3_(CC)_3_CN),^6^ 2-cyanoindene,^7^ cyanopyrenes,^8,9^ and cyanocoronene^10^ have recently been detected in the interstellar medium (ISM) by radioastronomy. Simple organic nitriles are also integral to understanding the planetary atmosphere of Titan, the largest moon of Saturn.^11^ The CN functional group introduces an inherently large dipole moment, which confers intense rotational transitions. A large fraction of the 330 molecules that have been detected in the ISM contain the nitrile functionality.^12,13^ Each of these nitrile-containing species may serve as a tracer molecule for its parent species, *e.g.*, benzonitrile for benzene, or 1- and 2-cyanonaphthalene for naphthalene, which cannot be readily detected by radioastronomy due to lack of a permanent dipole moment.^14^ Laboratory kinetics studies of the barrierless reaction between CN and C_6_H_6_ confirmed that benzonitrile formation is feasible in the low-temperature environment of the ISM, providing experimental evidence that CN-substituted species can likely be generated directly from their parent aromatic compounds.^14^ There are no analogous detections of cyano-substituted tracer molecules for nitrogen-containing heteroaromatic species currently detected in the ISM, *e.g.*, pyridine, pyrimidine, or pyridazine. These aromatic heterocycles have been extensively sought in the ISM, but not yet detected.^15–19^

Obtaining evidence that directly indicates the presence of any *N*-containing heterocycle in an extraterrestrial environment would be a significant advance in astrochemistry, and the relationship between heterocycles and open-chain organic nitriles is a plausible route to understanding their interstellar chemistry.^5^ It is not known whether the formation of heterocycles in the ISM would occur via a “top-down” process involving degradation of larger polycyclic ring systems or a “bottom-up” process involving cyclization of ubiquitous carbon-chain species. While less thermodynamically favorable than their heterocyclic counterparts, numerous open-chain organic nitriles (and isonitriles) of various chain lengths and levels of unsaturation have been detected in the ISM.^5,6,20–29^ Recent laboratory studies have shown that *N*-heterocyclic compounds can be generated from known interstellar components via electric discharge, UV photolysis, and pyrolysis techniques.^30,31^ Using an electric discharge reaction of benzene with N_2_, numerous nitrogen-containing hydrocarbons of various types, *e.g.*, 1*H*-pyrrole, benzonitrile, and acrylonitrile, were generated and identified using microwave spectroscopy.^31^ One of the pyridine isomers (C_5_H_5_N) generated in that study,^31^ (*E*)-1-cyano-1,3-butadiene. has been reported in TMC-1 as part of the GOTHAM survey,^32^ though this detection has recently been questioned.^33^ Although the laboratory spectrum for the vibrational ground state of the isomer, (*Z*)-1-cyano-1,3-butadiene, has been reported,^31,34,35^ this species was not identified in either of the astronomical observations.^32,33^ One of those studies^33^ provided tentative identification of a different C_5_H_5_N isomer, 2-cyano-1,3-butadiene, in TMC-1 using the previously reported laboratory rotational spectrum.^36^ Experimental studies demonstrated that pyridine can be formed by reaction of CN radical with 1,3-butadiene or by reaction of cyanovinyl radical (HC=CHCN) with vinyl cyanide (acrylonitrile, C_2_H_3_CN).^30^ The ISM detection of many varieties of open-chain nitriles and the laboratory results of their reaction chemistry suggest open-chain isomers are part of a complex set of chemical reactions in this environment and may provide insights into the search for their corresponding missing *N*-containing heterocycles.^15–19^

Our group maintains an interest in synthesizing, measuring the rotational spectra, and reporting the transition frequencies and spectroscopic constants of organic nitriles to facilitate their radioastronomical searches, particularly nitrile isomers of pyridine (Figure 1).^34,36–39,46^ Previously, we reported the synthesis^46^ and analysis of the ground-state rotational spectra of three open-chain pyridine isomers shown in Figure 1: (*Z*)-1-cyano-1,3-butadiene (***Z*** **–1**), (*E*)-1-cyano-1,3-butadiene (***E*** **–1**), and 4-cyano-1,2-butadiene (**2**).^34^ These three cyanobutadiene isomers share structural similarities with known astronomical molecules vinyl cyanide (C_2_H_3_CN),^21^ cyanodiacetylene (HC_4_CN),^22^ and cyanoallene (C_3_H_3_CN).^28^ While (*Z*)-1-cyano-1,3-butadiene, (*E*)-1-cyano-1,3-butadiene, and 4-cyano-1,2-butadiene are predicted to be 24, 23, and 40 kcal/mol higher in energy than pyridine (B3LYP/6-311+G­(2d,p)), respectively, they are more polar (μ = 3.7–4.7 D) than pyridine (μ = 2.215(1) D)^42^ and thus more readily detectable by radioastronomy. Mishra *et al.* showed experimentally that the flash vacuum pyrolysis of *trans*-3-pentenenitrile, another potential ISM molecule, preferentially produced (*Z*)-1-cyano-1,3-butadiene as the major product among several open-chain nitriles.^35^ Interestingly, the more thermodynamically favorable pyridine molecule was not detected in either the electric discharge or flash vacuum pyrolysis experiments.^31,35^ In addition to their possible relevance to the interstellar chemistry of ring systems, (***Z*** **–1**) and (***E*** **–1**) could also serve as tracers for the parent diene, 1,3-butadiene, a molecule that has been searched for but not successfully detected in the ISM.^47^ The similarities in structure to known interstellar molecules, relationships to fundamental species, and intense rotational transitions make the cyanobutadiene isomers attractive ISM targets for detection by radioastronomy and motivates the study of their laboratory rotational spectra.

**Figure 1:**
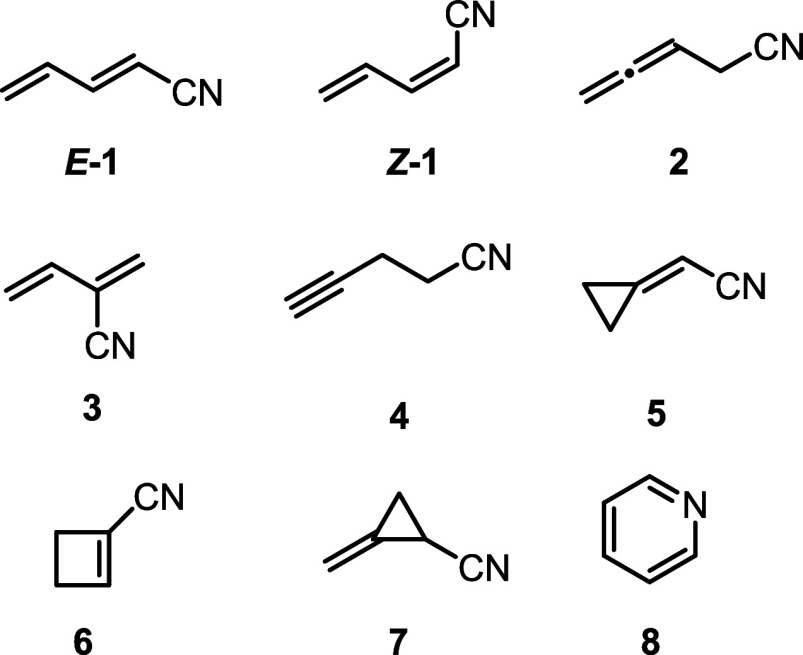
C_5_H_5_N isomers that have been studied by rotational spectroscopy: (*E*)-1-cyano-1,3-butadiene (***E*** **–1**),^31,34^ (*Z*)-1-cyano-1,3-butadiene (***Z*** **–1**),^31,34,35^ 4-cyano-1,2-butadiene (**2**),^34^ 2-cyano-1,3-butadiene (**3**),^36^ (cyanomethylene)­cyclopropane (**4**),^37^ 1-cyanocyclobutene (**5**),^38^ 4-cyano-1-butyne (**6**),^39^ 1-cyano-2-methylenecyclopropane (**7**),^40^ and pyridine (**8**).^41–45^

Of the four cyanobutadiene isomers that we have studied by rotational spectroscopy, *s-trans*-(*Z*)-1-cyano-1,3-butadiene^34^ and 2-cyano-1,3-butadiene^36^ each exhibit an isolated ground vibrational state and two low-lying, coupled vibrationally excited states. A detailed analysis of the coupled states was reported for 2-cyano-1,3-butadiene^36^ but not for *s-trans*-(*Z*)-1-cyano-1,3-butadiene. The current work presents our analysis of the Coriolis-coupled dyad of excited vibrational states (ca. 130 cm^–1^) of *s-trans-*(*Z*)-1-cyano-1,3-butadiene (Figure 2). These states have a predicted energy difference of less than 6 cm^–1^ and, as a result, their energy levels are heavily mixed. Several matching pairs of *a*- and *b*-type resonant transitions, as well as many formally forbidden, nominal-interstate transitions, are observed. Analysis of the spectra of these states yields a highly precise and accurate energy difference, as well as an accurate determination of spectroscopic parameters.

**Figure 2:**
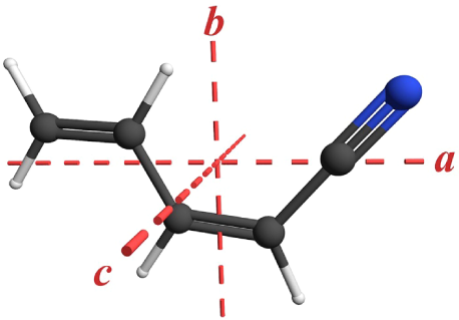
*s-trans*-(*Z*)-1-Cyano-1,3-butadiene (*C*_*s*_, C_5_H_5_N) structure with principal axes (μ_*a*_ = 3.3 D, μ_*b*_ = 2.3 D, MP2/6-311+G­(2d,p)).

## Computational and Experimental Methods

Geometry optimizations followed by anharmonic frequency calculations were carried out using Gaussian 16 through the WebMO interface.^48,49^ Computational predictions were completed using density functional theory (B3LYP/6-311+G­(2d,p)) and *ab initio* (MP2/6-311+G­(2d,p)) methods with “verytight” convergence criteria and an “ultrafine” integration grid. As described previously,^40^ the output files of the Gaussian calculation (available in the Supporting Information) are mislabeled as being in the III^*r*^ representation, but the constants are actually calculated in the I^*r*^ representation. An additional optimization and subsequent anharmonic frequency calculation were performed using a development version of CFOUR^50^ at the MP2/cc-pCVTZ level. Coriolis coupling terms ( $G_{a}^{19,27},G_{b}^{19,27},F_{ac}^{19,27}$ , and $F_{bc}^{19,27}$ ) were computed using the inertial tensor derivatives and cubic force constants predicted by the MP2/cc-pCTZ anharmonic frequency calculation in conjunction with equations derived from the Watson Hamiltonian treated by Van Vleck perturbation theory for vibration–rotational coupling.^51^

(*Z*)-1-Cyano-1,3-butadiene was synthesized as described previously.^46^ To reduce sample degradation, the neat liquid was kept in an ice–water bath during collection of the spectrum. The sample was maintained at a vapor pressure of 3 mTorr in a flow system at 0 to −50 °C. The broadband frequency spectrometer has previously been described.^52,53^ The complete spectrum from 130 to 375 GHz was obtained using automated data collection software over approximately 4 days with these experimental parameters: 0.6 MHz/sec sweep rate, 10 ms time constant, and 50 kHz AM and 500 kHz FM modulation in a tone-burst design.^54^ The least-squares fit and prediction of the rotational spectrum were obtained using Pickett’s SPFIT/SPCAT, and Kisiel’s Assignment and Analysis of Broadband Spectra (AABS) package was used to combine the 130–230 and 235–375 GHz experimental broadband segments.^55,56^ The PIFORM, PLANM, and AC programs were used for data analysis and for generating graphs and plots.^55,56^ A uniform frequency measurement uncertainty of 50 kHz was assumed for all transitions. The least-squares fitting files, as well as the computational output files, can be found in Supporting Information.

## Results and Discussion

### Summary of the Prior Ground-State Analysis

The ground-state potential energy surface of (*Z*)-1-cyano-1,3-butadiene is computed to display two low-energy conformations, but the structure of one of the conformers depends on the computational method employed (Figure 3). At the B3LYP/6-311+G­(2d,p) level of theory, a planar, *s-cis* conformer (*C*_*s*_) is computed to be an energy minimum on the potential energy surface. At the MP2/6-311+G­(2d,p) level, a nonplanar, *gauche* conformer (*C*_1_) (dihedral angle of 33°) is computed to be an energy minimum, while the *s*-*cis* conformer (*C*_*s*_) represents a low-energy transition state between two enantiomeric *gauche* conformers.^57^ Both methods predict a planar structure for the lower energy, *s-trans* conformer (Figure 3). The energy difference between the two low-energy conformers is slightly smaller on the MP2 surface (3.4 kcal/mol) than on the B3LYP surface (4 kcal/mol). In an earlier study, the ground-state rotational spectrum of the lower-energy *s-trans* conformation was observed (*vide infra*), but the spectrum of the higher-energy conformation (*s-cis* or *gauche*) was not.^34^ Dipole moments of the two conformers are comparable (*gauche*: μ_*a*_ = 3.0 D, μ_*b*_ = 2.3 D, μ_*c*_ = 0.3 D; *s-trans*: μ_*a*_ = 3.3 D, μ_*b*_ = 2.3 D, MP2), so the millimeter-wave rotational spectra should not be biased in favor of one conformer or the other. The Boltzmann population of the higher-energy conformer simply appears to be too small, in the temperature range of measurement (0° to −50°), to enable the identification of weak lines of this conformer amid the spectral density associated with transitions of the ground state and vibrationally excited states of the *s-trans* conformer. For the *s-trans* conformer, the experimental spectroscopic constants of the ground state can be treated via a distorted rotor Hamiltonian^34^ and used as a reference for treating the more complicated interactions in the vibrationally excited states.

**Figure 3:**
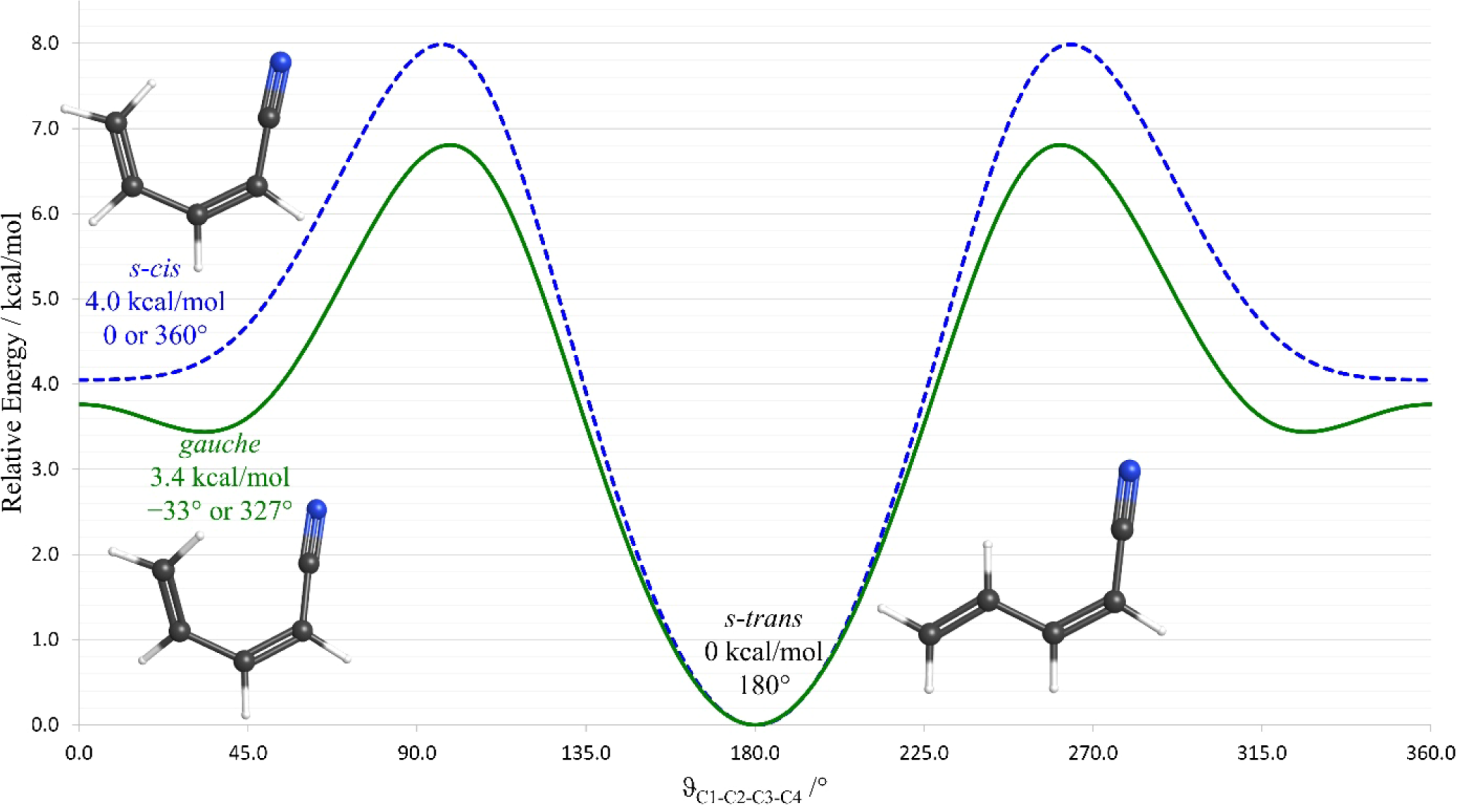
Computed conformational potential energy surface of (*Z*)-1-cyano-1,3-butadiene (B3LYP/6-311+G­(2d,p), blue dashed line; MP2/6-311+G­(2d,p), green solid line).

*s-trans*-(*Z*)-1-Cyano-1,3-butadiene (*C*_s_) is a prolate (κ = −0.91), asymmetric top with substantial dipole moments along the *a-* and *b-*principal axes (μ_*a*_ = 3.3 D, μ_*b*_ = 2.3 D, MP2/6-311+G­(2d,p)). Previously, 5495 ground-state transitions were measured in the 135–375 GHz region where intense R-branch series were observed as well as many Q- and weak P-branch transitions.^34^ We combined and least-squares fit the millimeter-wave data with 34 hyperfine-resolved microwave transitions from McCarthy *et al.* ^31^ and two from Mishra *et al.* ^35^ to a partial-octic, distorted-rotor quadrupole coupled Hamiltonian (σ_fit_ = 38 kHz error). No obvious signs of unaddressed Coriolis coupling were observed in the ground-state experimental spectroscopic constants (Table 1).^58^ These values were well-predicted (*obs. – calc.* < 16.5%) by an anharmonic frequency calculation at MP2/6-311+G­(2d,p). Predicted values of the octic centrifugal distortion constants are not available, so these constants are evaluated by comparison to those of similar molecules like the other cyanobutadiene isomers.^34,36^ Upon comparison with the other cyanobutadienes, the octic terms determined for *s-trans*-(*Z*)-1-cyano-1,3-butadiene do not appear to be unreasonable.

**Table 1:**
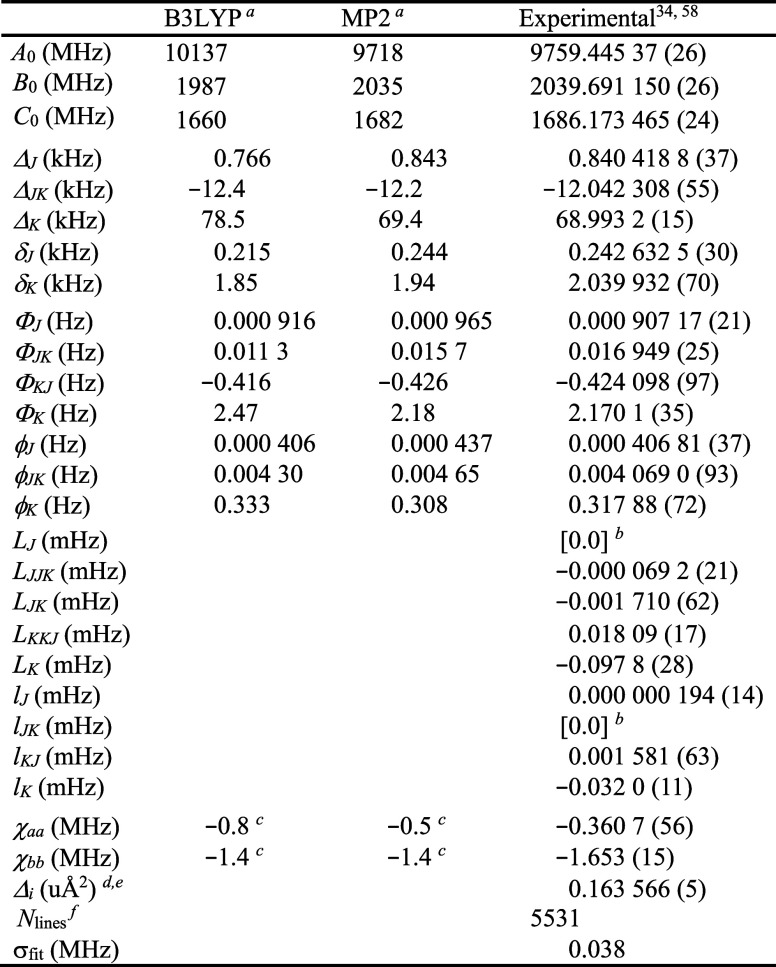
Spectroscopic Constants for the Ground Vibrational Stateof *s-trans*-(*Z*)-1-Cyano-1,3-butadiene (A-Reduced Hamiltonian, I^*r*^ Representation)

a Evaluated with the 6-311+G­(2d,p) basis set.

b Constants in square brackets are held fixed at the specified value.

c Constants converted from the field gradient axis system to the inertial moment axis system.

d Inertial defect, *Δ*_i_ = *I*_c_ – *I*_a_ – *I*_b_.

e Calculated using PLANM from the *B*_0_ constants.

f Number of fitted transition frequencies.

### Excited Vibrational States, ν_19_ and ν_27_

The vibrationally excited states of *s-trans*-(*Z*)-1-cyano-1,3-butadiene (Figure 4) include a dyad of Coriolis-coupled states (ν_19_ and ν_27_) near 130 cm^–1^ followed by a Coriolis-, Fermi-, and Darling-Dennison-coupled polyad of the two-quanta vibrational states (2ν_19_, ν_19_+ν_27_, and 2ν_27_) and along with fundamental ν_26_, near 250 cm^–1^. Due to the proximity of ν_18_ (310 cm^–1^) to the polyad, it is likely that neither can be adequately treated independently across the frequency range of this study. Our current analysis is limited to the lower-energy dyad of ν_19_ and ν_27_.

**Figure 4:**
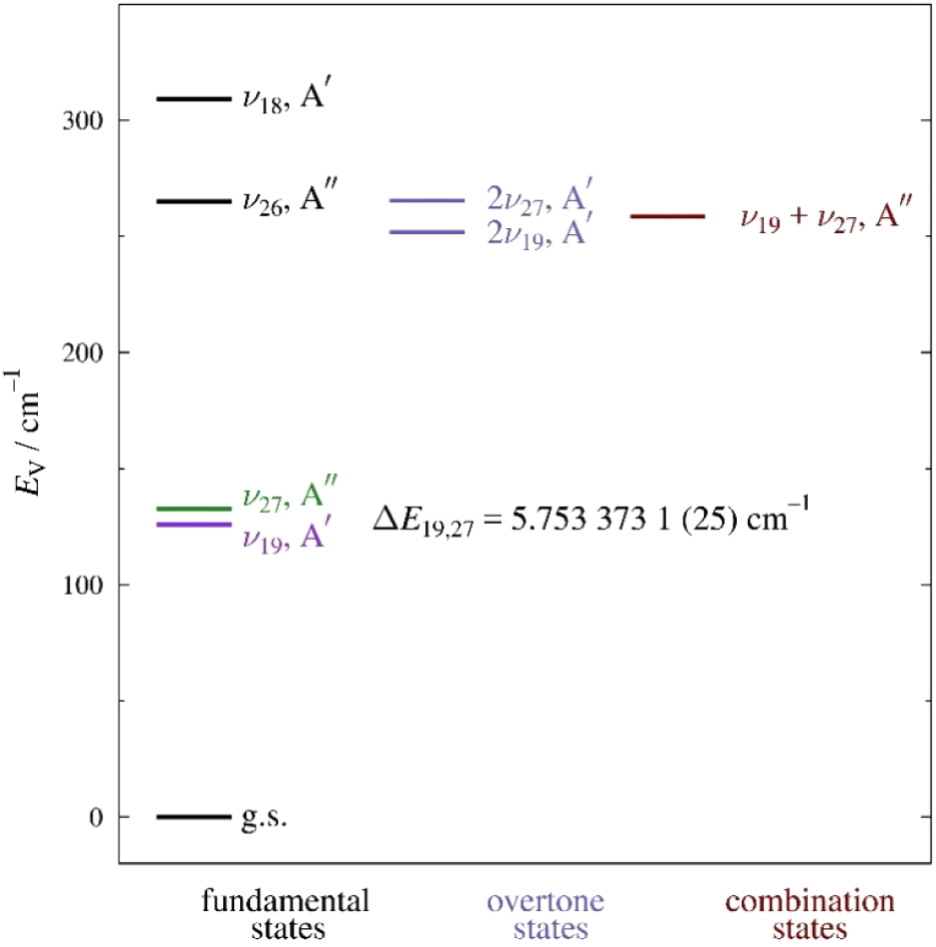
Vibrational energy levels of *s-trans*-(*Z*)-1-cyano-1,3-butadiene below 350 cm^–1^ from computed fundamental frequencies (MP2/6-311+G­(2d,p)). The value of Δ*E*_19,27_ results from the experimental perturbation analysis of ν_19_ and ν_27_ in this work.

The first vibrationally excited state, ν_19_ (A′, 126 cm^–1^, MP2), is an in-plane bending motion of the CN group. The second vibrational excited state, ν_27_ (A″, 133 cm^–1^, MP2), is an out-of-plane wag of the C=CH_2_ group. Several factors—the energy difference of these states (Δ*E*_19,27_ = 6.8 cm^–1^, MP2), the large values of *J* for the observed transitions across this frequency region (up to *J* = 110), and the relatively large magnitude of the predicted Coriolis ζ values—are all *a priori* indicators that the energy levels of these states will exhibit strong mixing. The computed *A*_0_–*A*_*v*_ vibration–rotation interaction constants are of equal magnitude and opposing sign (−81.8 MHz for ν_19_ and 86.8 MHz for ν_27_), suggesting they are absorbing unaddressed Coriolis coupling in the computational prediction. The transitions of these two vibrationally excited states are prominent throughout the spectrum and progress away from the ground-state bands in opposite directions (Figure 5). In the initial least-squares fit, predictions of the transitions for both vibrationally excited states were made from the computed vibration–rotation interaction constants and the experimental spectroscopic constants of the ground state. In the spectral region depicted in Figure 5, the typical oblate-type bands of R-branch transitions of the ground state and ν_27_ transitions progress toward higher frequency with increasing *J* and *K*_*a*_, while transitions for ν_19_ progress toward lower frequency. For ν_19_ and ν_27_, the R-branch bandheads begin as quadruply degenerate transitions at *K*_*a*_ = 0^+^/1^–^ (^*a*^R_0,1_, ^*b*^R_1,1_, and ^*b*^R_–1,1_), where all four transitions share the same value of *K*_*c*_. For ν_19_, transitions of the band shown lose degeneracy at *K*_*a*_ = 5^+^/6^–^, while for ν_27_, transitions lose degeneracy at *K*_*a*_ = 4^+^/5^–^. With increasing *K*_*a*_, both vibrational states eventually complete multiple turnarounds and form degenerate series (same *K*_*a*_, different *K*_*c*_) with the same value of *J*. Both *a-* and *b-*type, R- and Q-branch transitions were sufficiently intense to be observed for ν_19_ and ν_27_. Unlike the ground state, however, P-branch transitions were not sufficiently intense to be observed for either excited state.

**Figure 5:**
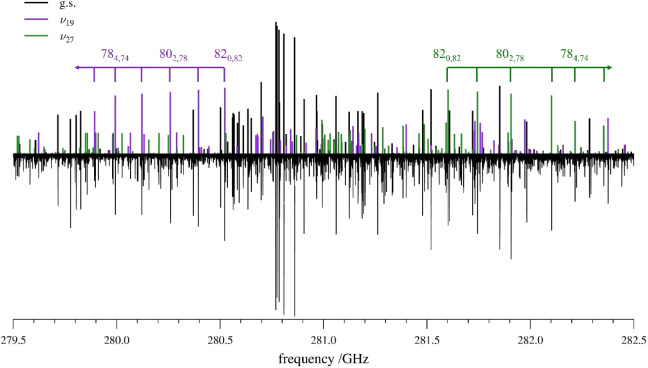
Predicted (top) and experimental (bottom) rotational spectra of *s-trans*-(*Z*)-1-cyano-1,3-butadiene from 279.5 – 282.5 GHz. Ground-state transitions are shown in black, while ν_19_ appears in purple and ν_27_ in green. Several transitions from the ^*a*^R_0,1_ branch bands are identified for ν_19_ and ν_27_. Many transitions belonging to other vibrational satellites are also visible in the experimental spectrum.

As expected, our initial least-squares fitting attempts of transitions for ν_19_ or ν_27_ quickly revealed that treatments involving single-state Hamiltonians were not adequate. As a result, a two-state Hamiltonian was employed to model the *a*- and *b*-axis Coriolis coupling, treating the rotational spectra of the in-plane vibration of the CN group and the out-of-plane twisting of the vinyl group in a manner similar to several previous studies involving coupled vibrational states.^37,38,53,59–66^ Initially, many centrifugal distortion constants needed to be held at their ground-state values and the Coriolis-coupling terms held at their computed values. As additional transitions were assigned, measured, and incorporated in the least-squares fit, additional spectroscopic constants were allowed to vary and Coriolis-coupling terms were included. Through this iterative process, we were able to achieve partial-octic fits of the spectroscopic constants for both states. The final data set includes 3415 transitions for ν_19_ and 3329 transitions for ν_27_, with both states covering *K*_*a*_″ ranges of 0–41 and *J*″ ranges 10–111 (Figure 6). The horizontal gaps in the plots are not due to unaddressed coupling or the quality of the spectroscopic constants derived from the least-squares fit; rather, they arise due to a small frequency gap in the hardware of the spectrometer. The final spectroscopic constants, given in Table 2, are quite well determined for both vibrational states. Constants that were unable to be satisfactorily determined were held constant at their corresponding ground-state values, as has been done previously.^37,38,53,59–66^

**Figure 6:**
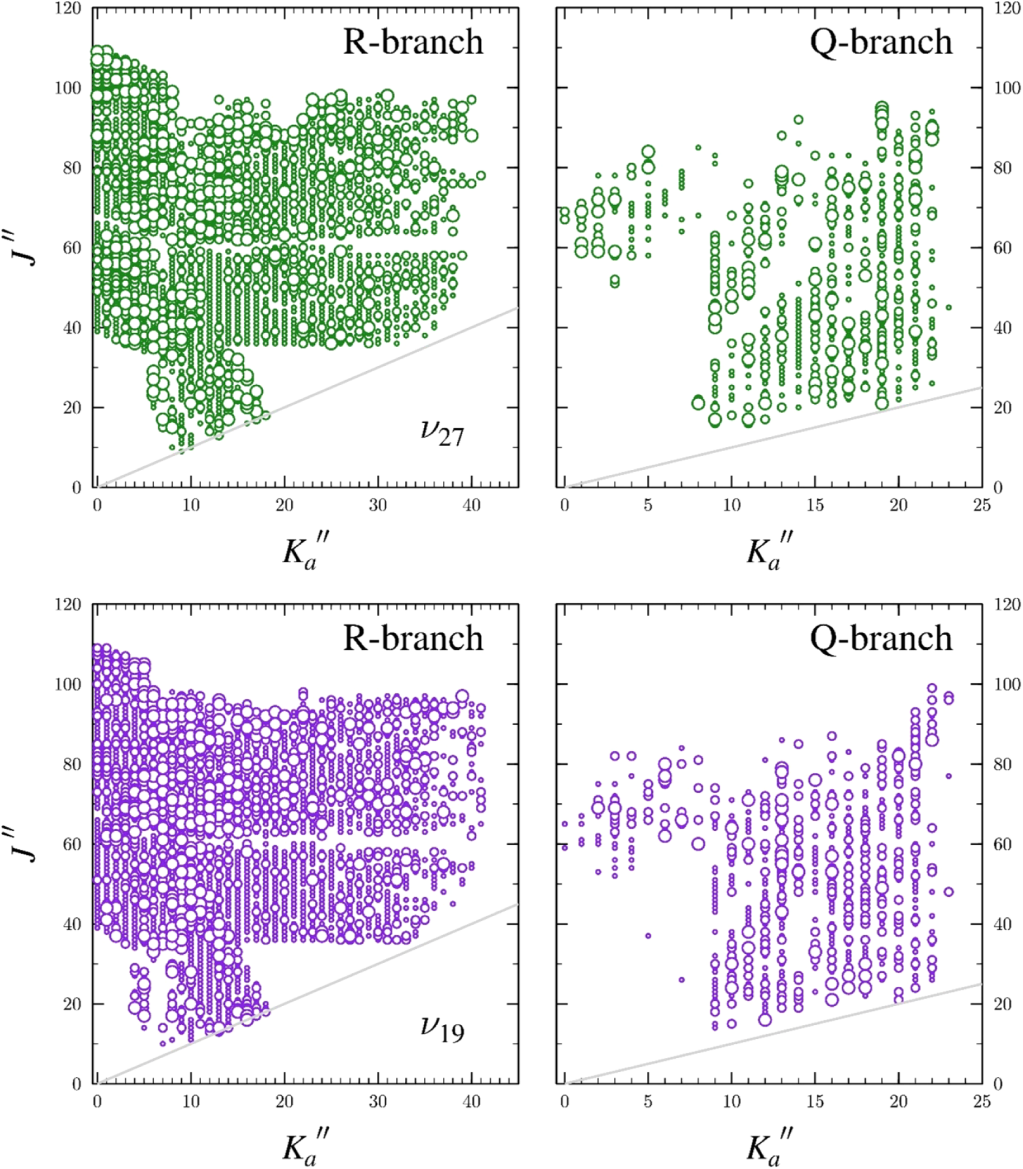
Data distribution plots for the coupled fit of measured transitions in the two lowest-energy excited vibrational states of *s-trans*-(*Z*)-1-cyano-1,3-butadiene: ν_19_ (purple) and ν_27_ (green). The size of the symbol is proportional to the value of |(*f*_*obs*._ – *f*_*calc*._)/*δf* |, where *δf* is the frequency measurement uncertainty, and all quotient values are smaller than 3.

**Table 2:**
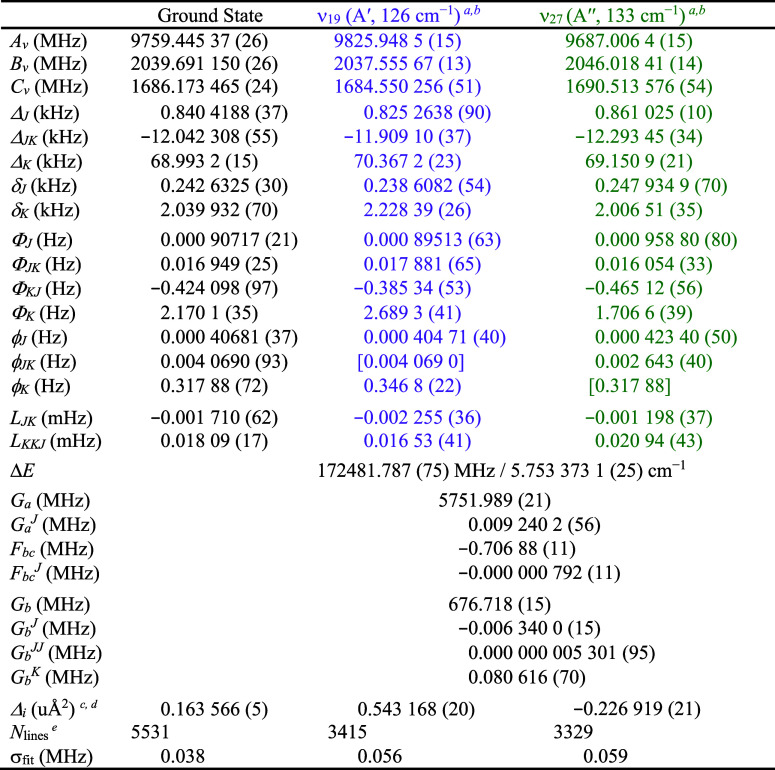
Spectroscopic Constants for Vibrationally Excited States ν_19_ and ν_27_ of *s-trans*-(*Z*)-1-Cyano-1,3-butadiene (A-Reduced Hamiltonian, I*^r^* Representation)

a Fundamental frequencies calculated using MP2/6-311+G­(2d,p).

b Centrifugal distortion constants in square brackets, or not shown, held constant at their ground-state values shown in Table 1.

c Inertial defect, *Δ* *_i_* = *I*_c_ – *I*_a_ – *I*_b_.

d Calculated using PLANM from the *B*_0_ constants.

e Number of fitted transition frequencies.

The largest change in the rotational constants of the vibrationally excited states, relative to the ground state, is observed for *A*_*v*_ (0.68% for ν_19_ and 0.75% for ν_27_). Almost all of the determined quartic and sextic distortion constants are quite similar to their corresponding ground-state values (< 27% difference), except for ϕ_*JK*_ and *L*_*JK*_ in ν_27_ (54% and 43%, respectively). We attempted to hold these constants fixed to the ground-state values but were not able to achieve a stable least-squares fit. Besides those two constants, the percent changes in the remaining constants are similar to changes that we observed previously in studies of analogous coupled dyads of cyano-substituent vibrations.^38,59–66^ The off-diagonal terms ϕ_*JK*_ in ν_19_ and ϕ_*K*_ in ν_27_ were the only two sextic terms that needed to be held at their ground-state values. We were required to vary a few octic centrifugal-distortion constants, determining values of *L*_*JK*_ and *L*_*KKJ*_ in both ν_19_ and ν_27_. We held the remaining octic terms fixed to their corresponding ground-state value. While the deviations between the ground-state, ν_19_, and ν_27_ centrifugal distortion constants are small, some of the deviations of the excited-state values, relative to the ground-state constant value, are nearly equal in magnitude and opposite in sign (Table 2). This condition is a signature that some of the centrifugal distortion constants may be influenced by untreated Coriolis coupling. Unlike our other studies of analogous coupled dyads, the σ_fit_ value of each vibrational state in *s-trans*-(*Z*)-1-cyano-1,3-butadiene is slightly above the assumed 50 kHz measurement uncertainty of the individual transition frequencies (ν_19_ = 55 kHz, ν_27_ = 58 kHz). In a similar fashion, the statistical uncertainty of the ground-state fit is also somewhat higher than typical in those works (σ_fit_ = 39 kHz), indicating the possibility of untreated interactions or centrifugal distortion in each of these least-squares fits. Attempts to reduce the statistical uncertainty of the least-squares fit by incorporating additional centrifugal distortion constants were unsuccessful. Given that the change in the statistical uncertainty between the ground state and excited dyad states is comparable to many of our prior works, the fact that the σ_fit_ value slightly exceeds 50 kHz is reasonable.

### Coriolis-Coupling Analysis

The symmetries of ν_19_ (A′, 126 cm^–1^) and ν_27_ (A″, 133 cm^–1^) allow *a-* and *b-* type Coriolis coupling to occur between these fundamental states. In the end, eight Coriolis-coupling terms were needed to address the intense mixing observed (*G*_*a*_, *G*_*a*_^*J*^, *F*_*bc*_, *F*_*bc*_^*J*^, *G*_*b*_, *G*_*b*_^*J*^, *G*_*b*_^*JJ*^, and *G*_*b*_^*K*^). Several *K*_*a*_ series between ν_19_ and ν_27_ are so strongly mixed that the quantum-number assignments of some transitions are unstable in SPFIT from iteration to iteration. With each iteration of the fit, some of these highly mixed energy levels from ν_19_ exchange quantum-number state labels of *J*, *K*_*a*_, *K*_*c*_, and ν with the perturbing energy levels in ν_27_. As a result, transitions originally labeled as in-state ν_19_ transitions were found to be labeled as ν_27_ transitions or as nominal interstate transitions. The corresponding simultaneous changes also occurred in transitions originally labeled as ν_27_, resulting in many transitions and series being rejected from the fit due to quantum-number label changes. While these transitions are correctly assigned, well-measured, and well-predicted, we excluded many of these transitions to achieve a more well-behaved and stable least-squares fit. A similar situation was observed and solution employed for the two lowest-energy fundamental states of propionitrile (ν_13_ and ν_21_)^67^ and for the two lowest-energy fundamental states of 2-furonitrile and 3-furonitrile (ν_24_ and ν_17_).^65,66^ The energy separations between the vibrationally excited states of these Coriolis-coupled dyads (6.207 024 6 (14) cm^–1^, 11.779 103 9 (36) cm^–1^, and 1.442 667 (10) cm^–1^, respectively) are small and comparable to the value determined in the current study. Similar to those works,^65–67^ adjusting the DIAG parameter in SPFIT was not able to resolve the state-labeling issue. The substantial state mixing is graphically represented in Figure 7, where the mixing coefficients of the ν_19_ and ν_27_ vibration–rotation energy levels are plotted by *J* and *K*_*a*_. In a figure of this type, the regions of significant mixing in each series typically appear as islands or ridges in the contour plot, and the height of the island is proportional to the degree of interaction between the states, *e.g.*, ν_22_ and ν_33_ of benzonitrile.^59^ Some resonances are seen with matching values of *J* that differ in *K*_*a*_ in a manner dependent on their respective selection rules. In each vibrational state, almost every *K*_*a*_ series from 0 to 21 includes several local perturbations and heavy energy-level mixing across our entire frequency range. The *J* > 90 region in *K*_*a*_ = 10–20 for both vibrational states is quite difficult to treat because of a large number of transitions with exchanging quantum-number labels. The contour plots of ν_19_ and ν_27_ have a more chaotic appearance than those of benzonitrile due to both substantial mixing that makes the state assignment ambiguous and the aforementioned instability in labels in SPFIT. The intense trapezoidal patch of mixing for ν_27_ (Figure 9) includes energy levels expected in its plot as well as those whose quantum numbers have switched between ν_19_ and ν_27_. Transitions with these *J* and *K* values correspond to several of the gaps and regions with larger circles (*obs*. – *calc*. between 50 and 100 kHz) that are evident in the data distribution plots in Figure 6. Above *K*_*a*_ = 21 in the current frequency range, the state-mixing decreases and the quantum-number instability is no longer an issue, corresponding to the very low mixing regions of the contour plots. Although the issues that arise from the intense state-mixing reduce the number of available transition frequencies to be included in the analysis, a well-determined set of spectroscopic constants was obtained for each vibrationally excited state due to the large number of remaining transition frequencies.

**Figure 7:**
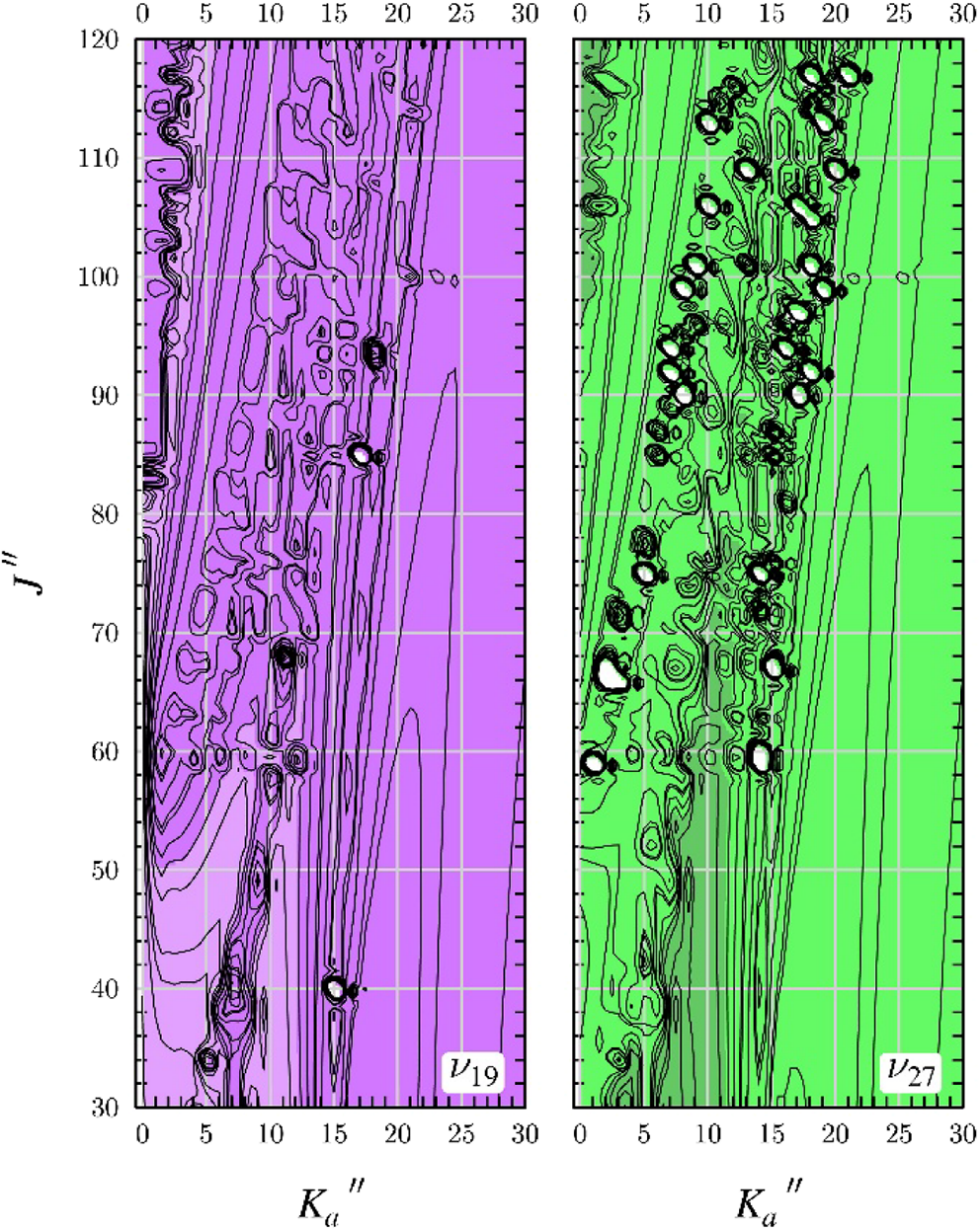
Contour plots depicting the coupling landscape between rotational levels in the ν_19_ (purple) and ν_27_ (green) vibrational states of *s-trans*-(*Z*)-1-cyano-1,3-butadiene. The mapped values are (1 – *P*_*mix*_) where *P*_*mix*_ is the mixing coefficient of a given vibration–rotation energy level. Resonances between levels in the two vibrational states are apparent as matching, similarly shaped islands along the horizontal direction (same *J*) but differing in the values of *K*_*a*_.

Numerous ν_19_ and ν_27_ transitions involved in matching *a-* and *b-*type resonances were included in the final least-squares fit. The resonance plots in Figure 8 show two correlated *K*_*a*_ series between ν_19_ and ν_27_ that have multiple pairs of matching resonances with Δ*K*_*a*_ = 2 selection rules. The most-displaced transitions in *K*_*a*_ = 5^*–*^ in ν_27_ and *K*_*a*_ = 7^+^ in ν_19_ are predicted to occur at *J*″ + 1 = 42 and are shifted by ∼19.1 GHz, with a predicted intensity too weak to be measured. A corresponding energy-level diagram of the same transitions involved in the *J*″ + 1 = 42 resonance from Figure 8 is shown in Figure 9. The intrastate transitions shown in this figure are not included in the data set because substantial intensity borrowing by interstate transitions reduces the intensity of the *J*″ + 1 = 42 transitions by ∼25 times that of the intensity of transitions at nearby *J* values within the same *K*_*a*_ series. Instead, the interstate transition intensity is almost the same as nearby intrastate transitions with proximate *J* values, and the intense mixing appears to transfer almost all of the intensity to the interstate transition. A second pair of *a*-type resonances with the same selection rules can be seen between *K*_*a*_ = 5^*–*^ and *K*_*a*_ = 7^+^ at *J*″ + 1 = 68 (Figure 8) and was included in the least-squares fit. While there would be a third matching *a*-type resonance visible at *J*″ + 1 = 76, that value in *K*_*a*_ = 5^–^ is assigned as an R_2,–2_ interstate transition and thus does not appear in a standard resonance plot. Both *J*″ + 1 = 76 transitions are included in the data set, however, and correspond to mirrored-frequency perturbations.

**Figure 8:**
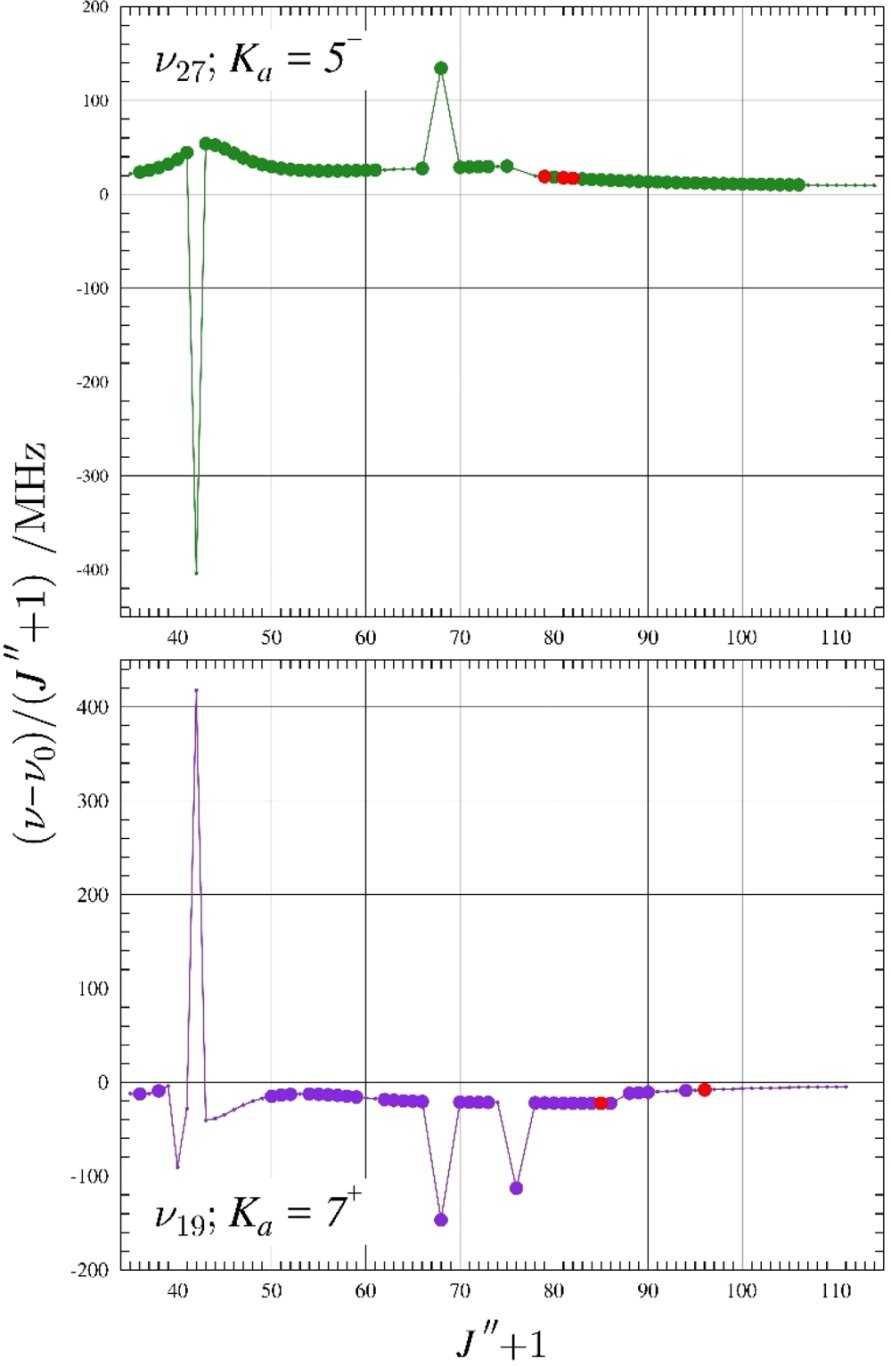
Resonance plots for *s-trans*-(*Z*)-1-cyano-1,3-butadiene showing the *K*_*a*_ = 7^+^ series for ν_19_ and *K*_*a*_ = 5^–^ series for ν_27_. Both observed resonances conform to the Δ*K*_*a*_ = 2 selection rules for *a*-type resonances. The plotted values are frequency differences between excited-state transitions and their ground-state counterparts. Measured transitions are represented by filled circles: ν_27_ (purple) and ν_17_ (green). Circles represent data with |(*f*_*obs.*_ – *f*_*calc*._)/*δf* | < 3 while red circles represent data with |(*f*_*obs.*_ – *f*_*calc*._)/*δf* | ≥ 3. Predictions from the final coupled fit are represented by a solid, colored line.

**Figure 9:**
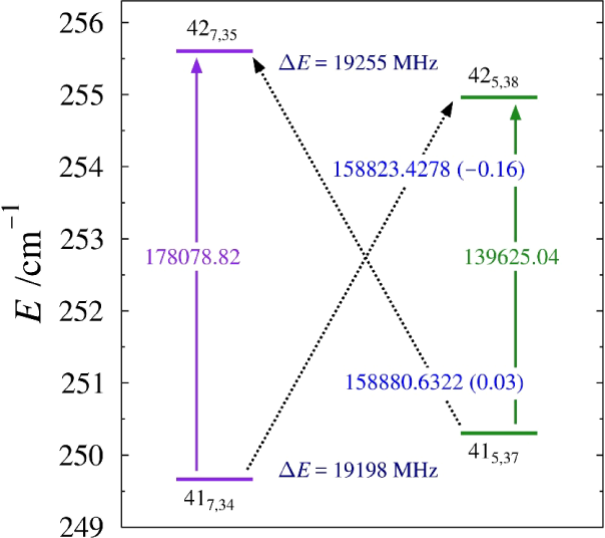
Energy diagram depicting a representative matched pair of nominal interstate transitions between the ν_19_ (purple) and ν_27_ (green) vibrational states of *s-trans*-(*Z*)-1-cyano-1,3-butadiene. Standard ^*a*^R_0,1_ transitions within vibrational states are denoted by vertical arrows with corresponding predicted frequencies. The diagonal, dashed arrows indicate nominal interstate transitions that are formally forbidden but enabled as a result of rotational-energy level mixing. Values printed on each of the arrows are the corresponding transition frequency (in MHz) with its *obs. – calc.* value in parentheses. The energy separation Δ*E* is given for each pair of strongly interacting rotational energy levels.

Figure 10a depicts several superimposed series of resonance plots, showing many progressions of *a*- and *b*-type resonances that increase in magnitude with *J*. The most-perturbed transition shown in Figure 10a is in *K*_*a*_ = 8 at *J*″ + 1 = 61; it is displaced from its unperturbed frequency by nearly 275 GHz. The most-perturbed transition estimated by this set of spectroscopic constants is in the *K*_*a*_ = 18^*–*^ series in ν_19_ and is shifted by 310 GHz (*J*″ + 1 = 95). Frequencies perturbed by such magnitudes, like those in *K*_*a*_ = 11–14 (Figure 10a), were completely outside of the 130–375 GHz frequency region and were unable to be observed, much less included in the data set. In many of these cases, we were instead able to observe and include the corresponding coupling-allowed, nominal interstate transitions due to almost complete intensity borrowing from the resonant intrastate transitions. Due to the magnitude of the resonances, a second plot limiting the *y*-axis to 500 MHz (the window of transitions we were able to observe) is shown in Figure 10b. The transition with the largest displacement that we managed to measure and include in the least-squares fit was shifted from its unperturbed frequency by 7.3 GHz. A broad undulation, which typically appears as an obvious, wave-like pattern in each *K*_*a*_ series, is obscured by even the smallest resonances in *s-trans*-(*Z*)-1-cyano-1,3-butadiene. The current set of spectroscopic constants can be used to reveal that identifiable frequency perturbations from the Coriolis coupling are present as early as *J*″ + 1 = 14 in *K*_*a*_ = 5, at a calculated frequency of 48.6 GHz.

**Figure 10:**
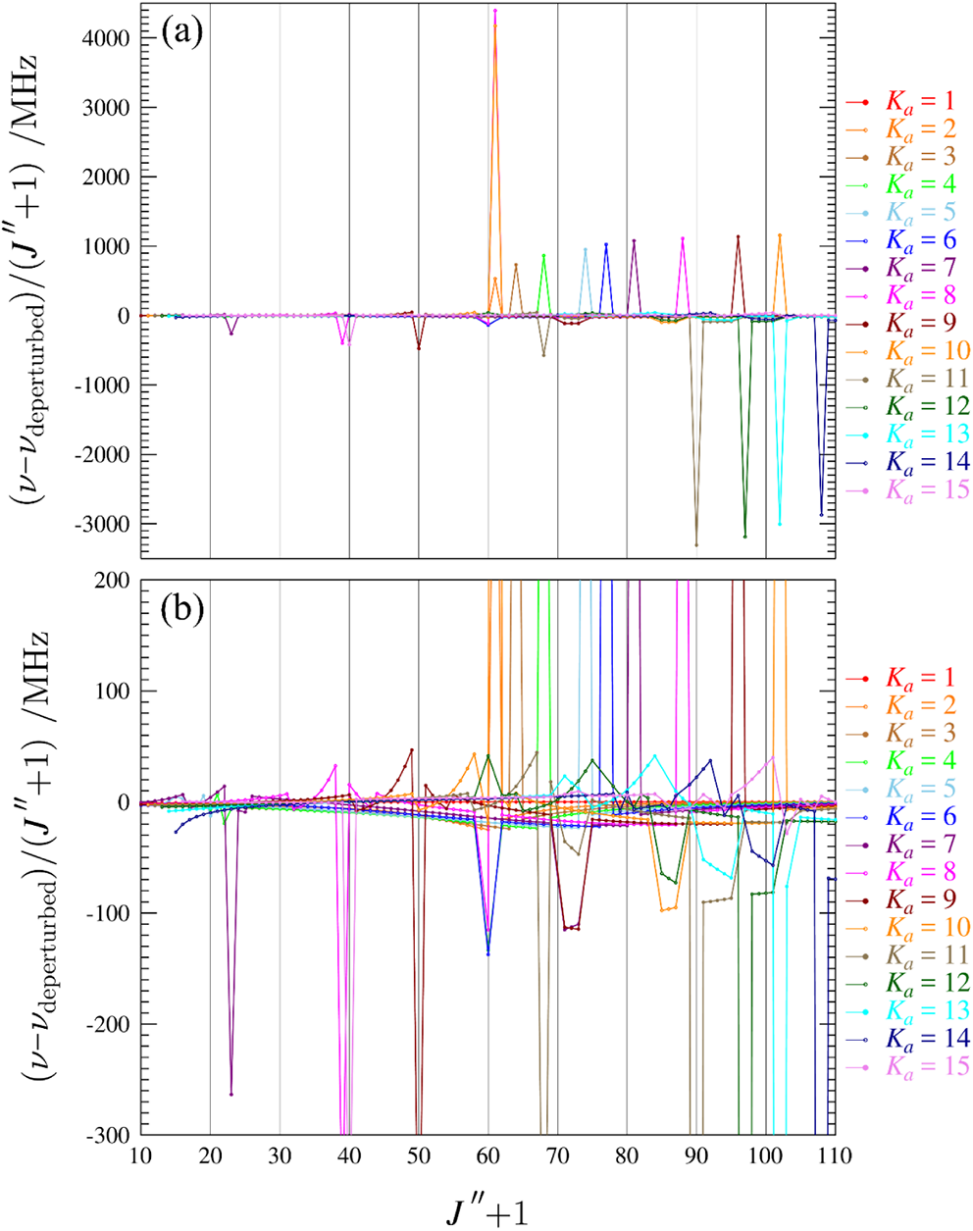
(a): Superimposed resonance plots of ν_19_ for ^*a*^R_0,1_ *K*_*a*_^–^ series from 1 to 15 for *s-trans*-(*Z*)-1-cyano-1,3-butadiene. Measured transitions are omitted for clarity, but they are indistinguishable from the plotted values on this scale. The plotted values are frequency differences between perturbed excited-state transitions from a deperturbed frequency, scaled by (*J*″ + 1). (b): Superimposed resonance plots with *y*-axis scaled to the frequency region observed in the current study.

The Coriolis-coupling analysis was used to determine a precise vibrational energy difference, Δ*E*_19,27_ = 5.753 373 1 (25) cm^–1^, between ν_19_ and ν_27_ (Table 3). Including transitions from local resonances where energy crossings occur, as well as many symmetry-allowed interstate transitions that change rotational and vibrational quantum numbers, contribute to the precision in the determination of the energy separation. The B3LYP-predicted energy difference of 6.1 cm^–1^ is an excellent estimate of the experimental value, and the MP2/6311+G­(2d,p) value is predicted to be only slightly higher (6.8 cm^–1^) (Table 3). Both of these values are in better agreement than the MP2/cc-pCVTZ value (9.0 cm^–1^). All of the B3LYP and MP2 calculations are in good agreement with the experimental Coriolis ζ values (Table 3), with the MP2 calculations showing slightly better agreement. Largely resulting from the vibrational-state symmetries not allowing Coriolis-coupling along the *c*-axis, all MP2-predicted values of *C*_0_ *– C*_19_ and *C*_0_ *– C*_27_ are in excellent agreement with the corresponding experimental values. Along the *a*- and *b*-axes, however, the computed and experimental vibration–rotation interaction constants are less reliable. There may be residual, untreated coupling absorbed by both the computed and experimental rotational constants. Taking the average of the vibration–rotation interaction constants for ν_19_ and ν_27_ brings all theoretical and experimental values into much closer agreement (Table 3).

**Table 3:** Experimental and Computed Vibration–Rotation Interaction Constants, Coriolis-Coupling Constants, and Energy Difference for Excited Vibrational States ν_19_ and ν_27_ of *s-trans*-(*Z*)-1-Cyano-1,3-butadiene

	Experimental	B3LYP^a^	MP2^a^	MP2^b^
*A*_0_ *– A*_19_ (MHz)	*–*66.503 2 (15)	*–*82.1	*–*81.8	11.4
*B*_0_ *– B*_19_ (MHz)	2.135 47 (14)	*–*0.027	0.90	2.61
*C*_0_ *– C*_19_ (MHz)	1.623 209 (69)	1.1	1.5	1.59
*A*_0_ *– A*_27_ (MHz)	72.438 9 (15)	86.3	86.8	*–*7.61
*B*_0_ *– B*_27_ (MHz)	*–*6.327 27 (15)	*–*4.1	*–*5.0	*–*6.60
*C*_0_ *– C*_27_ (MHz)	*–*4.340 111 (71)	*–*3.6	*–*4.1	*–*4.12
$\frac{\left(A_{0}-A_{19}\right)+\left(A_{0}-A_{27}\right)}{2}$ (MHz)	2.967 8 (22)	2.1	2.5	1.9
$\frac{\left(B_{0}-B_{19}\right)+\left(B_{0}-B_{27}\right)}{2}$ (MHz)	*–*2.095 90 (20)	*–*2.1	*–*2.1	*–*2.0
$\frac{\left(C_{0}-C_{19}\right)+\left(C_{0}-C_{27}\right)}{2}$ (MHz)	*–*1.358 45 (10)	*–*1.3	*–*1.3	*–*1.3
$\left|G_{a}^{19,27}\right|$ (MHz)	5751.989 (21)	6420^c^	6030^c^	6050^c^
				6070^d^
$\left|F_{bc}^{19,27}\right|$ (MHz)	0.706 88 (11)			0.293^e^
$\left|G_{b}^{19,27}\right|$ (MHz)	676.718 (15)	828^c^	800^c^	800^c^
				805^d^
$\left|F_{ac}^{19,27}\right|$ (MHz)				1.17^e^
$\left|\zeta_{19,27}^{a}\right|$ (MHz)	0.294 586 (97)^c^	0.329	0.309	0.310
$\left|\zeta_{19,27}^{b}\right|$ (MHz)	0.165 830 (55)^c^	0.203	0.196	0.196
Δ*E*_19,27_ (cm^–1^)	5.753 373 1 (25)	6.1	6.8	9.0

a Evaluated with the 6-311+G­(2d,p) basis set.

b Evaluated with the cc-pCVTZ basis set.

c Calculated by the approximate relationship $G_{a}^{19,27}=2\,\;\;A_{0,exp}\,\;\zeta_{19,27}^{a}$ .

d Calculated by $G_{a}^{19,27}=(\left(\omega_{A}+\omega_{B}\right)/\sqrt{\omega_{A}\omega_{B}})\;{\;A}_{e}\,\;\zeta_{19,27}^{a}$ .

e Calculated using the inertial tensor derivatives and cubic force constants from an anharmonic frequency calculation at the MP2/cc-pCVTZ level.

Computed values for Coriolis-coupling constants $G_{a}^{19,27}$ and $G_{b}^{19,27}$ (Table 3) were determined using either an approximate relationship involving the Coriolis zeta constants $\left(G_{a}^{19,27}=2\,\;\;A_{0,exp}\,\;\zeta_{19,27}^{a}\right)$ or the properties predicted by an MP2/cc-pCVTZ anharmonic frequency calculation, involving harmonic frequencies, equilibrium rotation constants, and Coriolis zeta constants $\left(G_{a}^{19,27}=(\left(\omega_{19}+\omega_{27}\right)/\sqrt{\omega_{19}\omega_{27}})\;A_{e}\,\;\zeta_{19,27}^{a}\right)$ . The $G_{a}^{19,27}$ and $G_{b}^{19,27}$ MP2/cc-pCVTZ values predicted by both the approximate equation and the slightly more accurate approach are nearly indistinguishable ( $G_{a}^{19,27}$ 6050 vs. 6066 MHz; $G_{b}^{19,27}$ 800 vs. 805 MHz; Table 3). Importantly, the two values are much closer to each other than they are to the experimental values, validating the use of the simplified equation as a reasonable approximation of these constants for initial values in the least-squares fitting process. The higher-order Coriolis-coupling constants $F_{ac}^{19,27}$ and $F_{bc}^{19,27}$ were calculated from the inertial tensor derivatives and cubic force constants from an anharmonic frequency calculation at the MP2/cc-pCVTZ level, using equations derived from the Watson Hamiltonian treated by Van Vleck perturbation theory for vibration–rotational coupling.^51^ These calculations use the methodology described in a preliminary fashion in the cited thesis, which will be the subject of a future detailed description. The computed and experimental values of $F_{bc}^{19,27}$ (0.293 MHz vs. 0.706 88 (11) MHz, respectively) are the same order of magnitude, though the computed value is smaller by more than a factor of 2. This is most likely an indication that the level of theory used to perform the anharmonic frequency calculation is inadequate or that the experimentally determined value of $F_{bc}^{19,27}$ is not physically meaningful, perhaps because $F_{bc}^{19,27}$ is absorbing Coriolis-coupling that should have been addressed by other Coriolis-coupling terms. The ability to determine a value for $F_{bc}^{19,27}$ is attributable to the more intense coupling along the *a*-axis, and thus more observed transitions with large frequency perturbations and nominal interstate transitions due to the *a*-axis coupling. These factors provide more *a*-axis coupling information to the least-squares fit, necessitating the inclusion of more Coriolis-coupling terms to account for the interaction, and resulting in the ability to fit $F_{bc}^{19,27}$ . $F_{ac}^{19,27}$ was not determinable from the transition data set, despite having a larger predicted value than $F_{bc}^{19,27}$ . Efforts were made to find the best set of Coriolis-coupling terms to model the data by trying various combinations of centrifugal distortion and Coriolis-coupling constants. Though it is possible that there are sets of spectroscopic constants that would provide more physically meaningful experimental values $F_{ac}^{19,27}$ and $F_{bc}^{19,27}$ , the least-squares fit presented in this work is the best that could be obtained from the current data set. Despite these limitations, the experimental determination and computational estimate of $F_{bc}^{19,27}$ provides an important benchmark.

## Conclusion

Cyanobutadiene isomers (C_5_H_5_N) are molecules of current interest in astrochemistry, in the context of both planetary atmospheres and the interstellar medium. The laboratory rotational spectrum of *s-trans*-(*Z*)-1-cyano-1,3-butadiene has been studied in the millimeter-wave region. The ground vibrational state is “isolated”: it does not exhibit coupling with other vibrational states, and the rotational spectrum is straightforwardly modeled as a simple distorted rotor. The two lowest-lying vibrationally excited states are strongly coupled. Analysis of the rotational transitions of ν_19_ and ν_27_ with the employed Hamiltonian accounts for all of the observed transition frequencies from 130 to 375 GHz for these states. This analysis is possible despite the intense perturbation of many transitions due to Coriolis coupling caused, in part, by the small energy difference between these fundamental states, Δ*E*_19,27_ = 5.753 373 1 (25) cm^–1^. The incorporation of over 3300 transitions in each of the coupled vibrational states enabled the determination of eight Coriolis-coupling terms between these states. The strong state mixing causes an instability in the quantum-number labels of the energy levels in the least-squares fitting program, which, as a practical matter, results in an inability to include all of the available data in the analysis. Fortunately, transitions that are included in the least-squares fit data set are involved in the broad curvature of *K*_*a*_ series, in the sharp local resonances, and in the coupling-allowed, formally interstate transitions. These data provide sufficient information to define Coriolis-coupling terms for vibrationally excited states ν_19_ and ν_27_. The necessity to exclude many transitions did not hinder the least-squares fitting or prediction of the rotational spectra of these vibrationally excited states.

## Supplementary Material





## Notes

Computational output files (PDF)
Least-squares fitting output files (ZIP)
