# Supplementary material for: The 130 to 375 GHz Rotational Spectroscopy of s-trans-(Z)‑1-Cyano-1,3-butadiene (C5H5N): Analysis of the Lowest-Energy Vibrationally Excited Dyad (ν19 and ν27)
Source: J Phys Chem A. 2025 Sep 4;129(37):8630–42. doi: 10.1021/acs.jpca.5c04066 (PMC12503384; doi:10.1021/acs.jpca.5c04066)
Supplement: Supplementary file 1 [file jp5c04066_si_001.pdf]

Supporting Information

The 130 to 375 GHz Rotational Spectroscopy of *s-trans*-(*Z*)-1-Cyano-1,3-butadiene (C_5H_5N): Analysis of the Lowest-Energy Vibrationally Excited Dyad (ν_{19} and ν_{27})

P. Matisha Dorman, Brian J. Esselman, Andrew N. Owen,
R. Claude Woods,* Robert J. McMahon*

Department of Chemistry, University of Wisconsin–Madison, Madison, Wisconsin 53706, USA

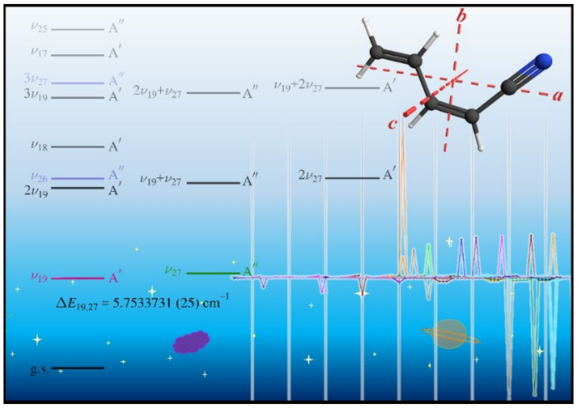

* Corresponding authors.
E-mail addresses: rcwoods@wisc.edu (R.C. Woods)
robert.mcmahon@wisc.edu (R.J. McMahon)

**Table of Contents**	**Page**
B3LYP/6-311+G(2d,p) geometry optimization and anharmonic frequency calculation for *s-trans*-(*Z*)-1-cyano-1,3-butadiene – Gaussian 16	Separate file
B3LYP/6-311+G(2d,p) geometry optimization and anharmonic frequency calculation for *s-cis*-(*Z*)-1-cyano-1,3-butadiene – Gaussian 16	Separate file
MP2/6-311+G(2d,p) geometry optimization and anharmonic frequency calculation for *s-trans*-(*Z*)-1-cyano-1,3-butadiene – Gaussian 16	Separate file
MP2/6-311+G(2d,p) geometry optimization and anharmonic frequency calculation for *s-cis*-(*Z*)-1-cyano-1,3-butadiene – Gaussian 16	Separate file
MP2/6-311+G(2d,p) geometry optimization and anharmonic frequency calculation for *gauche*-(*Z*)-1-cyano-1,3-butadiene – Gaussian 16	Separate file
MP2/6-311+G(2d,p) conformational scan – Gaussian 16	Separate file
MP2/cc-pCVTZ geometry optimization *s-trans*-(*Z*)-1-cyano-1,3-butadiene – CFOUR	Separate file
MP2/cc-pCVTZ VPT2 anharmonic frequency calculation *s-trans*-(*Z*)-1-cyano-1,3-butadiene – CFOUR	Separate files
Least-squares fitting output files for the vibrational ground state of *s-trans*-(*Z*)-1-cyano-1,3-butadiene	Separate file
Least-squares fitting output files for coupled-state fit of ν_{19} and ν_{27} of *s-trans*-(*Z*)-1-cyano-1,3-butadiene	Separate file
